# A Study of Strain-Driven Nucleation and Extension of Deformed Grain: Phase Field Crystal and Continuum Modeling

**DOI:** 10.3390/ma11101805

**Published:** 2018-09-23

**Authors:** Ling-yi Kong, Ying-jun Gao, Qian-qian Deng, Zhi-rong Luo, Yu-jiang Lu

**Affiliations:** 1 Guangxi Key Laboratory for the Relativistic Astrophysics, Guangxi College Key Laboratory of Novel Energy Materials, School of Physics Science and Engineering, Guangxi University, Nanning 530004, China; 15567206166@163.com (L.-y.K.); 13768113812@163.com (Q.-q.D.); robot-163@163.com (Z.-r.L.); 15778115265@163.com (Y.-j.L.); 2 Institute of Physics Science and Engineering Technology, Yulin Normal University, Yulin 537000, China

**Keywords:** nucleation and growth, strain-driven, Phase Field Crystal, continuous model

## Abstract

The phase-field-crystal (PFC) method is used to investigate migration of grain boundary dislocation and dynamic of strain-driven nucleation and growth of deformed grain in two dimensions. The simulated results show that the deformed grain nucleates through forming a gap with higher strain energy between the two sub-grain boundaries (SGB) which is split from grain boundary (GB) under applied biaxial strain, and results in the formation of high-density ensembles of cooperative dislocation movement (CDM) that is capable of plastic flow localization (deformed band), which is related to the change of the crystal lattice orientation due to instability of the orientation. The deformed grain stores the strain energy through collective climbing of the dislocation, as well as changing the orientation of the original grain. The deformed grain growth (DGG) is such that the higher strain energy region extends to the lower strain energy region, and its area increase is proportional to the time square. The rule of the time square of the DGG can also be deduced by establishing the dynamic equation of the dislocation of the strain-driven SGB. The copper metal is taken as an example of the calculation, and the obtained result is a good agreement with that of the experiment.

## 1. Introduction

Grain boundary structures in nanometer- and sub-micro-sized polycrystalline materials during plastic deformation processes have attracted tremendous attention many years motivated by their wide potential used in technologies [1,2]. Many researches for grain boundary sliding (GBS) have been achieved in conventional polycrystalline materials. Migration of grain boundary (GB) is a fundamental mechanism in recrystallization and grain growth [1,3]. At present, the research of the motion of the grain boundary dislocation (GBD) is an active study field in microstructure evolution, in particular, in the collective motion of the GBD coupling with the applied stress or strain [2]. Recent research has focused on the recognition that many GBs in crystalline materials can couple to applied shear stresses and are moved by them in a manner similar to dislocation glide [3,4,5,6,7,8,9,10]. The coupling can be responsible for the stress–induced grain growth in nanocrystalline materials and influences the nucleation of new grain during recrystallization. There are two main modes of nanograin growth [11,12,13], i.e., the shear-coupled migration of the GB, and nanograin rotation, and these two modes are usually coupled [14]. The dominant mode of the operation of these two modes depends on grain size [14]. Now researchers not only still pay more attention to motion of the GBs coupling with stress or strain, but also to the GB pre-melting (GBPM) [15,16,17,18,19] and the grain growth driven by stress [20,21,22,23] at high temperature. With the rapid development of computer technology, the roles of computer numerical simulation technology in materials are more and more prominent. Computer numerical simulation technology, real experimental observation and theoretical model analysis are the same important and are known as three great scientific research methods since the 21th century. Now the technique of the numerical computational simulation has been extensively used to many fields and can make up for the shortcoming [24] in real experimental observation. Molecular dynamic (MD) [24,25,26,27,28] has been used to simulate the migration of the GB and the stress–driven grain growth. Although much success is obtained by MD simulations, a weakness [29] of the MD approach is that the applied strain rate in simulations is likely to deviate by several orders from the actual results. Traditional phase field approach is also used to simulate the elastic deformation-driven grain growth in copper polycrystals [30], while it is difficult to describe the details of the migration of the GB [31,32] on nanoscale.

Elder [33,34] et al. proposed a phase field crystal (PFC) model based on density functional theory in recent years, which can well simulate evolution of microscopic structure of nano- and poly-crystalline materials on diffusive time scale and on atomic resolution scale, and the applied strain rate in simulations is a good agreement with the actual results. Therefore, it has a great advantage in simulating the evolution of the GB migration and grain growth on nanoscale. The PFC model is uniquely suited to study grain growth under applied strain because it captures atomic motion over diffusive timescales. Atomic resolution is required to resolve the lattice continuity across the GB and, as we will show, the diffusive timescales are necessary to observe grain growth. In the past few years the PFC has been successfully used to many fields of the research [35,36,37,38,39,40,41,42,43,44,45,46,47,48]. Although there have been several studies [48,49,50,51,52,53,54,55] focused on cooperative dislocation movement (CDM) [46,51,52,55] of the GB and grain growth by the PFC approach, so far, the mechanism of the strain-driven CDM of the SGB split from the original GB [13] and also of the strain-driven nucleation and growth of the deformed grain with localized strain energy are still unclear. The author has previously studied the CDM of the strain-driven migration of the GB using the PFC method, the contents of which include new grain generation through the splitting of the GB of the bicrystal [46], and two stage processes [52] of the annihilation of the dislocations at high temperature, and pre-melting of the GB with proliferation [56] and annihilation of the dislocation, and also the rotation [55] of the dislocation. However, there is still a lack of research of microscope kinetic on the nucleation and growth of the deformed grain with the localized plastic flow. In this paper, based on PFC simulation of the GB dislocation motion and combined with continuous model, we explore the SGB migration and CDM under diviatoric deformation, and analyze the phenomenon of the localized plastic flow. Furthermore, we study the mechanism of the biaxial strain-driven nucleation of the deformed grain, and as well as the growth of the deformed grain with higher localization strain energy, and deeply reveal the law of the growth of the deformed grain by establishing the dynamic equation of the strain-driven dislocation movement.

## 2. Model and Method

In PFC model, a periodic order parameter [33] is defined by the local-time-averaged atomic density *ρ*. The dimensionless free energy functional [33,44] *F* of the system is given as

(1) $$F=\int f(\rho (x(1+\varepsilon ),y(1-\varepsilon )))dV=\int [f(\rho (x,y))+E_{ext}(\varepsilon ,x,y)]dV\;$$

where *f*(*ρ*) is a local free energy density functional [34], which can be written as

(2) $$f(\rho (x,y))=\left[\frac{1}{2}r\rho^{2}+\frac{1}{4}\rho^{4}+\frac{1}{2}\rho {\left(1+\nabla^{2}\right)}^{2}\rho \right]\;$$

where *r* is a parameter relate to temperature, $\nabla^{2}$ is the Laplace operator. *E_ext_* is the energy of the system changing by external force [2,41,44], be written as

(3) $$E_{ext}(\varepsilon ,x,y)=V_{ext}\cdot \rho \;$$

where *ε* is the strain. An analytical expression of the atomic density can be obtained from Equation (1) under the minimum of free energy. Then, the atomic density for the two-dimensional triangular structure in the one-mode approximation [33,34] can be written as

(4) $$\rho =A_{T}[\cos(qx)\cos(qy/\sqrt{3})-\cos(2qy/\sqrt{3})/2]+\rho_{0}\;$$

where *ρ*_0_ is the average value of *ρ*, the wave vector $q=\sqrt{3}/2$. The evolution of the atomic density of the conserved field variable is described by the nonlinear Cahn-Hilliard dimensionless Equation [34,56] as below

(5) $$\frac{\partial \rho }{\partial t}=\nabla^{2}(\frac{\delta F}{\delta \rho })=\nabla^{2}[r\rho +\rho^{3}+{\left(1+\nabla^{2}\right)}^{2}\rho +V_{ext}]\;$$

where *V_ext_* given in Reference [56]. Equation (5) can be solved by using the semi-implicit Fourier spectral method [56,57,58].

For simplicity, a two-dimension (111) plane in an FCC lattice is chosen and used as a simulation sample system. In order to more carefully study the strain-driven migration of GB which cause the extension of deformation zone and the localized strain energy, in this paper, a bi-crystal system with the small-angle symmetric tilt grain boundary (STGB) is designed in the two-dimension equivalent (111) plane, which is gotten by using the atomic density distribution formula (4). In the present work, we follow the Reference [29] to choose a square domain with *L_x_* × *L_y_* = 512Δ*x* × 512Δ*y* as a sample for simulating, and to apply the periodic boundary condition. The expression of the tilt angle *θ* for the STGB and the grain orientational direction are given in detail in reference [46]. Here we set *θ* = ±4° to get a sample with bicrystalline structure. The parameters for the preparation of samples are set as: *r* = −0.10, *ρ*_0_ = −0.180. Before the external strain applying to the sample, it is needed for relaxation of the sample and the relaxing time will last for 1 × 10^5^-time steps, in order that the system can reach equilibrium state. After the relaxation, we can get the higher temperature sample with a bi-crystal structure.

**Figure 1:**
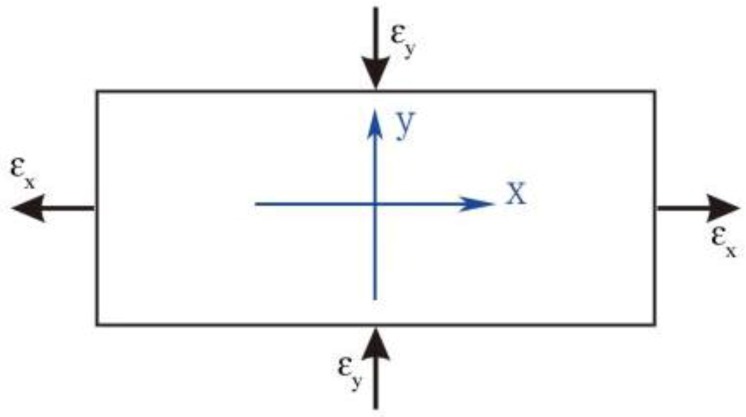
Exerting biaxial strain on the sample for deviatoric deformation: A tension is along *x* direction with *ε_x_*, and along *y* direction is a compression with *ε_y_*.

We perform the deformation for sample shown in Figure 1 and employ the periodic boundary condition [48,56] on it.

The applying strain method in the PFC model under constant volume condition [48] can be easily and directly used by periodic boundary condition without designing a complex modulation compensation function [29] for boundary condition. Therefore, for conveniences, we use the constant volume condition in order to exert the biaxial strain effect on the sample for the deviatoric deformation in this work. Since the constant strain rate is applied to all atoms, the deformation state becomes the affine deformation state, and the periodic boundary conditions can be easily used as a boundary condition. According to Reference [48] for the tensile deformation, we perform the deformation simulation for sample shown in Figure 1 by using the constant volume condition [34] with the periodic boundary conditions [48]. The strain has the form of $\varepsilon =\varepsilon_{x}=\varepsilon_{y}=\dot{\varepsilon }n\Delta t$, where the strain rate is set to be $\dot{\varepsilon }=6\times 10^{-6}/\Delta t$, and Δ*t* is time step (*ts*) and *n* is the number of the time step. They are both in the same time step Δ*t* = 0.5 in the semi-implicit Fourier spectral method in our PFC simulation for density field evolution and strain rate loading (The Reference [34] reports that in real space operation, Δ*t* = 0.0075, the operation speed is very slow, and the efficiency of calculation is very low). Total evolution times are 20,000-time steps. According to the constant area (volume) condition, we have

(6) $$S=\Delta x\Delta y=\Delta x^{\prime }\Delta y^{\prime }\;$$

where Δ*x* and Δ*y* are the initial grid sizes, Δ*x*’ and Δ*y*’ are the grid sizes under deformation. At the *n*th time steps of the simulation, the grid sizes in the *x* and *y* direction have the formula [48,52,56,59], respectively, as below

(7) $$\Delta x^{\prime }=\Delta x(1+\varepsilon )=\Delta x(1+n\dot{\varepsilon }\Delta t)\;$$

(8) $$\Delta y^{\prime }=\Delta y/(1+\varepsilon )\approx \Delta y(1-\varepsilon )=\Delta y(1-n\dot{\varepsilon }\Delta t)\;$$

**Figure 2:**
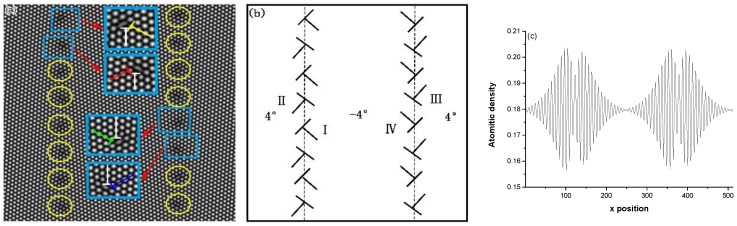
(**a**) The dislocations structure in the Symmetric tilt grain boundary (STGB) in bicrystal system designed by PFC method. Inset: the dislocation configuration of the blue box area; (**b**) Schematic of the oblique dislocation arrangement (here the vertical dislocation arrangement is ignored, in order to highlight the slip motion of dislocations, which would be really helpful to understand the following results) in the STGB with four kinds of the dislocation I, II, III and IV; (**c**) The average spatial distribution of the order parameter of the atomic density inside the sample projects to *x* axis.

In this case, a tension is along *x* direction, while along *y* direction is a compression. More detail about the numerical computation of the deformation simulation can be seen in Reference [48,56,59]. The sample with the GB and the dislocation arrangement made by PFC is shown in Figure 2.

The elastic properties of the two-dimensional triangular state can be obtained by considering the energy costs for deformation from the equilibrium state. The free-energy density associated with deviatoric deformation can be calculated by considering modified forms of Equation (4), i.e., *ρ*(*x*(1 + *ε*), *y*(1 − *ε*)). In such calculation *ε* represents the dimensionless deformation. The results of the calculations to determine the strain energy for the two-dimensional system given in Reference [34] can be written as

(9) $$F_{dev}=F_{\min}^{}+F_{el}=F_{\min}^{}+[C_{11}-C_{12}]\cdot \varepsilon^{2}\;$$

and the elastic constants are gotten as

(10) $$C_{11}/3=C_{12}=C_{44}=\alpha /4\;$$

These results are consistent with the symmetries of a two-dimensional triangular system, i.e., *C*_11_ = *C*_12_ + 2*C*_44_. Since the STGB of the bicrystals is flat and no curvature, the STGB migration driven by deviatoric deformation is of the CDM. The strain energy can be written as *f_d_* = Δ*F_dev_* = Δ*E_str_* = *m*(*C*_11_ − *C*_12_)·*ε*^2^ [30,60,61], *m* is a constant.

In Figure 2a, the yellow circle areas indicate the place where there are dislocations in the GB, while the blue boxes indicate the magnified areas with the dislocation region and give the configuration of the dislocations. Since these vertically arranged dislocations in Figure 2a only reflect the nature of the difference in misorientation of the GBs, here, the slip motion of the vertically arranged dislocations of the grain boundaries is not obvious, but the slip motion is only expressed by the oblique rows of the dislocations in Figure 2b. In order to highlight the dislocation slipping, here the vertical dislocations are ignored. When paying attention to the annihilation of the GB dislocation, the vertically arranged dislocations cannot be ignored, because it also participates in the annihilation. The Reference [52] gives an analysis of this annihilation process.

## 3. Simulation Result and Discussion

### 3.1. Deformaed Grain Nucleation

**Figure 3:**
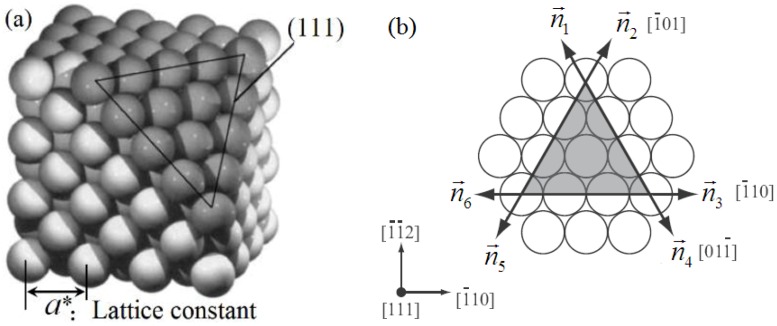
Three-dimensional (3D) atomic lattice of face-centered cubic (FCC) structure (**a**), the index of direction of atomic lattice arrangement in (111) plane of FCC structure [62] in two dimensions (**b**).

Here we consider a two-dimension system of the sample. The crystalline symmetry of the sample in two dimensions here is equivalent to the {111} family of planes in fcc lattice or the {0001} family of planes in a hcp lattice. These close packed planes and the subsequent glide directions compose the primary slip systems in many common types of ductile, metallic crystals [58]. Using the fcc lattice as a reference, application of shearing in this geometry results in glide along a <110> direction within a {111} slip plane, as shown in Figure 3. For simplicity, the two-dimensional (111) plane in a fcc lattice of metal, for example, copper is chosen and used as a simulation system. Schematic illustrations of the initial GBs and dislocation arrangement of the sample (here the vertical dislocation arrangement is ignored) are given in Figure 2b. Each GB is composed of eight dislocations with two sets of Burgers vector which are arranged in a straight line [59,61]. Therefore, there are two types of the GB dislocations (i.e., lattice dislocation [61]) locating in the same GB. The dislocations in contrary GB are with opposite Burgers vector. Hence there are four types of the GB dislocations in sample, which are denoted as I, II, III and IV, respectively, as shown in Figure 2b. The average spatial distribution of the atomic density is given in Figure 2c, which is a sum of the average density values over *y*-axis plotted as a function of *x*. It can be seen that the order parameter amplitude at the GB is lower than that of the surrounding of the GB. This indicates that the order degree of the GB is lower, owing to the dislocations [46,62,63].

**Figure 4:**
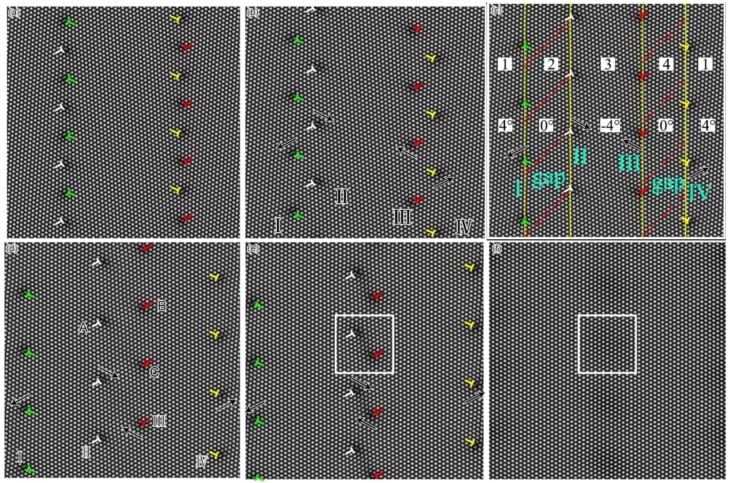
The snapshots of migration and annihilation of the dislocation of the SGB in sample under the applied strain. Here the vertically arranged dislocations are ignored in the present paper in order to highlight the slip motion of dislocations, which has some different from that in previous publication such as Reference [46,52,56,62]. In these figures, the arrows indicate the directions of the slip motion of the dislocations, and the gap indicates the region with red slashes between the SGBs where the deformed grain is. The numbers 1, 2, 3, 4 represent respectively four grains, and 4°, 0°, −4° represent the misorientation of the grains, respectively. White box area in the figure shows the dislocation annihilation and the generation of the complete crystal. (Although previous studies reported similar results, they did not reveal these phenomena from the dynamics of deformed grains. This is the biggest difference between the present work and the previous results [46,52])

Here we exert the deviatoric deformation on the sample, and observe the evolution of the CDM of the GB, and also the nucleation and extension of the deformed grain. Figure 4a–f shows the separating movement of the STGB dislocations and the new grain (i.e., the deformed grain) nucleation and growth in the sample under the deformation (seeing the Figure 4c, the gap region with red slash is the region between the SGBs, which is also the deformed grain with orientation 0°). At the beginning, the system is the bicrystal structure in Figure 4a. Under the biaxial strain, owing to activate the slip system, the original STGBs are split into two SGBs to generate a gap (i.e., the new grain generates) in between the two SGBs, then the deformed grain nucleates as shown in Figure 4a,b. (Although previous studies reported similar results, they did not reveal these phenomena from the dynamics of deformed grains. This is the biggest difference between the present work and the previous results [46,52]). Since the same type of the dislocations of the STGB moves collectively at the same speed and along the same direction, they are in the same straight arrangement in whole process of the movement. The arrangement of these dislocations is considered as the SGB [61,62], and denoted respectively as I, II, III and IV, which constitute a four-grain system. The orientation angles of the four grains are respectively 4°, 0°, −4°, 0°, shown in Figure 4c, where the grain with orientation 4°and −4° is the original grains(OG) denoted as 1 and 3, while the grain gaps with orientation 0° are the new deformed grains (DG) denoted as 2 and 4. According to the vector expression of the dislocation at the GB in Reference [61,62], the direction index of Burgers vectors of dislocation in GB are shown in Figure 3b. The migration mechanism of the CDM through gliding, decompounding and annihilating under the applied strain, can be used to reveal the nature of the deformation of nanocrystalline (NC) material [64,65,66,67,68,69,70], creep [71], super-plasticity [11] at high temperature.

### 3.2. The Localized Strain Energy and Localized Plastic Flow

**Figure 5:**
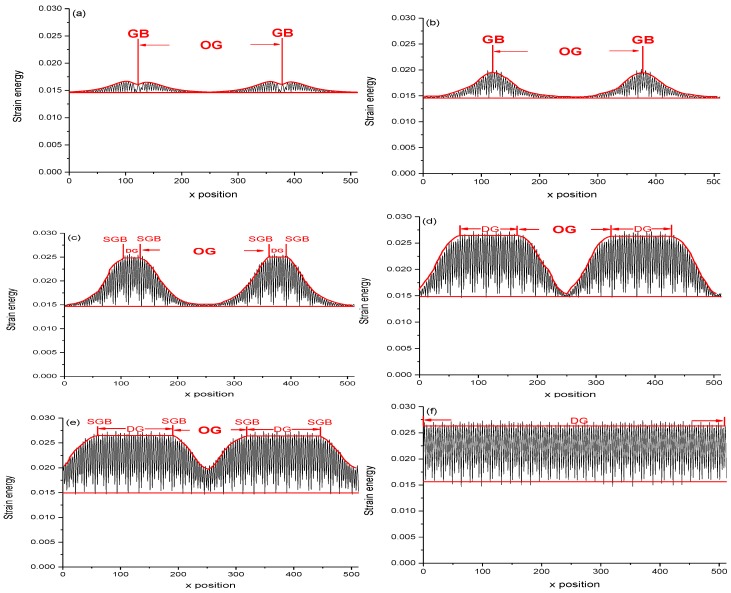
Snapshots of the spatial distribution of the strain energy inside the sample by the PFC simulation is projected to *x* axis for different strain, which is a sum of the density values over *y*-axis plotted as a function of *x*. The red line shows the profile of the spatial distribution of the strain energy. The DG is the width of the deformed grain in these pictures, and the OG is the width of the original grain in these pictures. Strain: (**a**) 0.000, (**b**) 0.0294, (**c**) 0.0516, (**d**) 0.0582, (**e**) 0.0664, (**f**) 0.0732.

In order to more clearly understand the strain state of the newly generated deformed grain, the spatial distribution of the strain energy is calculated using the energy Equation (1) of the PFC. The snapshot of spatial distributions of the strain energy projecting to the *x* axis for the sample at different amounts of the strain, which is a sum of the density values over *y*-axis plotted as a function of *x*, are shown in Figure 5, in which the area surrounded by the red line corresponds to the increase of the strain energy under the action of the strain. The red horizontal line in Figure 5 is the reference height of the energy before the biaxial strain exertion. The energy above the reference height, which is added to the system, is the elastic strain energy in the early stage of deformation, while that is the plastic strain energy in the later stage of the deformation. As shown in Figure 5a without the external applied strain, the strain energy distribution for the original grain (OG) in the sample is concentrated on the GB and its surrounding, and its energy is about 0.0170 greater than that 0.015 inside grain, which is due to the strain energy of the dislocation stored. It can be seen that when the applied strain exerts on the sample, the peak of the strain energy distribution at the GB rises up in Figure 5b. This indicates that the localized strain energy is firstly concentrated on the GB. At this time, the width of the peak of the energy distribution becomes widened in Figure 5b,c. This indicates that the dislocation of the GB also moves along the direction perpendicular to the GB by gliding in Figure 4, and results in the formation of the ensembles of the mobile lattice dislocation [64,65,66], which are the carriers of the plastic flow [66,70,71,72]. The area swept by the ensembles of the mobile lattice dislocation (i.e., plastic flow) [71] will get more strain energy by changing the orientation of the crystal lattice, as shown in Figure 5c,d. Such a separated process of the SGB with the ensembles of the mobile lattice dislocation results in plastic flow localization [65,73,74,75,76] (deformed band) in mechanically. This situation corresponds to the nucleation and growth of the new softened deformation band (grain) [73]. The band or the gap in between the two opposite SGBs separated can be considered as the new deformed grain with orientation angle 0° shown in Figure 4 marked by grain 2 and 4.

With the applied strain increasing, when the localization strain energy at the GB reaches the maximum value of about 0.026 shown in Figure 5c, the height of the peak of the energy no longer increases. At this time, the peak of the energy distribution of the curve for the GB begins to extend to both sides to form a higher platform of the energy, as shown in Figure 5c–e. The width DG of the platform in the energy distribution expresses the (band) width of the DG in Figure 5c–e, in which the height of the peak of the energy distribution is proportional to the amount of the localized strain energy in unit area in the DG. This indicates that the interior of the DG has accumulated more localized strain energy, and the highest strain energy density in the original GB site of the grain keeps a constant, which height is about 0.026 and is much higher than that about 0.015 inside the OG. There is an obvious dividing boundary between the DG and the OG in the localized strain energy distribution. As the area of the higher localization strain energy extends, finally, two higher energy platform regions meet and connect as shown in Figure 5e. At this time, the height of the overall energy platform of the sample increases from 0.015 up to 0.026, and the energy density is uniformly distributed in the whole sample to become the non-localized uniform strain energy. This process is that the area with the higher localized strain energy extends into the area with the lower strain energy, and the DG extends and consumes the OG with lower strain energy to become a single deformed crystal with higher uniform distribution of the strain energy, and the OG disappears as shown in Figure 5f.

It seems intuitive [73] that the strain energy would be reduced by the motion of the GBs towards the grain with higher strain energy, however, the simulation results of this works for the bicrystal with the applied strain shows that the SGB moves towards the OG with lower strain energy. The similar phenomenon is also presented by Tonks et al. [73]. In addition, it is found that the DG is softened by the applied deformation [30,72,73]. The softened DG, due to the plastic flow localization (i.e., the collective cooperation movement of the dislocations driven by the strain) can store more strain energy because it accommodates more of the applied strain than the hard OG [30,73]. The plastic flow localization usually occurs in the process of the deformation of nanocrystalline (NC) metal [66,67,68,69,70] and metallic glass [74,75,76]. For example, because NC copper is three times more resistant to deformation than coarse-grained copper, its deformation is homogeneous without apparent necking by a steady plastic flow until sample failure occurred [30], i.e., the materials displayed near-perfect elastic-plastic behavior [30]. Similar prefect elastic-plasticity in nano-materials is observed by other researchers [30,71,73], both in tension and compression. This behavior can be explained by high atomic diffusion and the small size of the grains, which will make it possible to activate superplastic-type mechanisms at room temperature [11,30,56,65,70,73].

**Figure 6:**
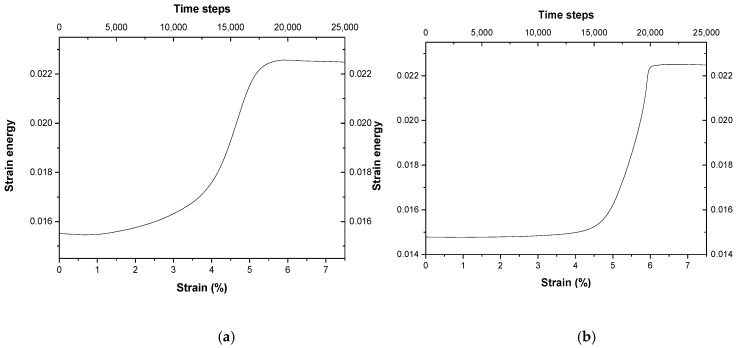
The localized strain energy change versus strain (**a**) in the zone of a rectangular region with red slash in Figure 4c; (**b**) in the zone of the original grain marked by grain 3 in Figure 4c.

Figure 6a shows the localized strain energy change versus time and/or strain in a rectangular region with red slash in Figure 4c. It can be seen that the strain energy curve for the GB region increases slowly during the beginning stage in the range of 0–2%. When the strain reaches the ranges of 2–4%, the energy increases gradually from 0.016 to 0.018. While the strain reaches the ranges of 4–5.5%, the energy increases quickly from 0.018 to 0.023. After that, the strain energy of the curve reaches a maximum value and keeps it at a constant 0.023. This indicates that the strain energy in unit area in the region swept by the SGB dislocations, obtains the maximum value. It can also be seen in Figure 6b that the energy in the region of the OG changes with time and/or strain increasing. When the strain *ε* is less than 4%, the energy hardly changes and stays at 0.015, which is lower than that in Figure 6a. This indicates that the internal strain energy of the OG is lower and is hardly accumulated under the strain. When the strain reaches the range of 4–6%, the strain energy inside the OG increases quickly. This indicates that the dislocation flow of the SGB of the DG enters into the interior of the OG and makes the change of the orientation of the local lattice of the grain and transforms the OG into the DG. When the strain reaches more than 6%, the strain energy of the region reaches the maximum and keeps it at a constant about 0.023. This indicates that the OG has completely transformed into the DG, and the strain energy of the whole system obtains a uniform distribution.

**Figure 7:**
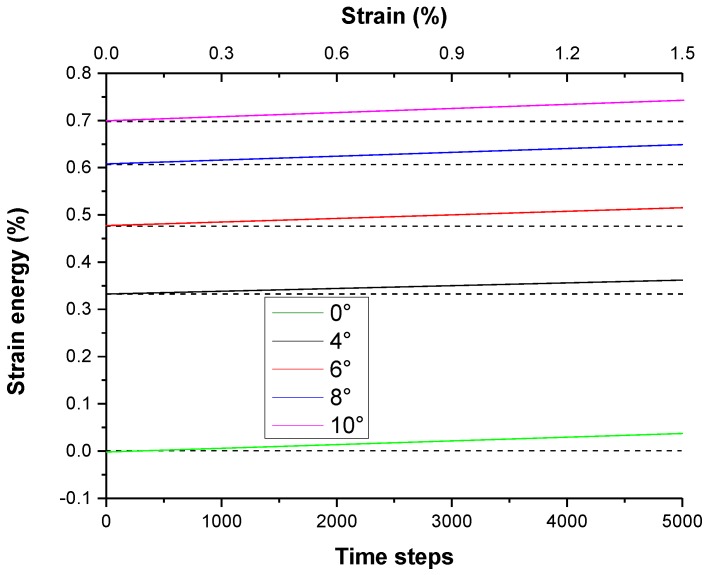
The increment percentage of the stored strain energy in deformed grain, which is calculated by using the PFC with the strain energy of single crystal as a reference point, changes with strain (%) or time steps for different misorientation *θ* of the GB.

As external strain is exerted on the sample, it can be seen that the strain energy of the different misorientations *θ* of the small-angle STGB increases with the strain of the deformation, as shown in Figure 7. The larger the misorientation *θ* of the GB, the more the strain energy stored in the GB are, which is due to more dislocations in the GB with large *θ*. Under the condition of the small *θ* <10° of the GB, the strain energy stored in the GB is approximately linear with the angle *θ* of the GB.

### 3.3. Extension of Localized Deformation Zone

**Figure 8:**
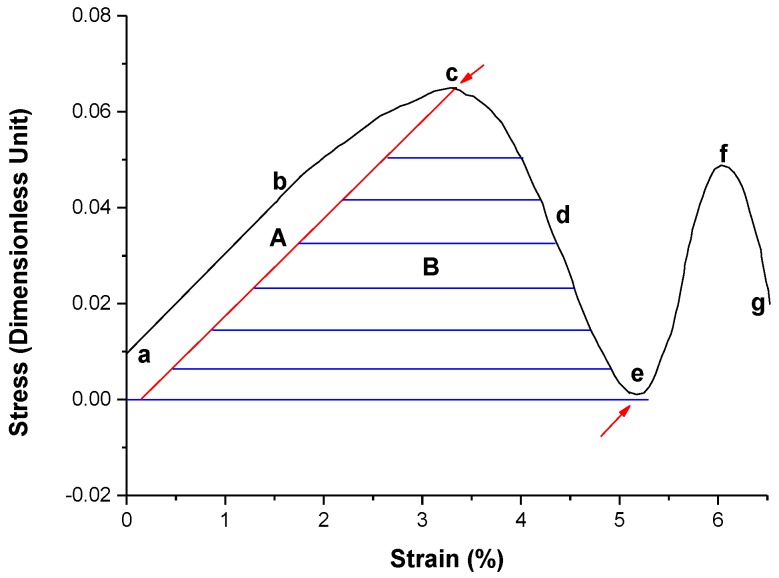
Stress-strain curve of the process of the deformation for the sample by the PFC simulation. The unit of the stress is the dimensionless.

The extension of the local deformed region corresponds to the growth process of the deformed grain. Figure 8 shows the average stress–strain (SS) curve of the sample system during the deformation. In the SS curve of the figure, the initial strain is not zero because the local internal stress exists in the GB dislocations in the sample and cannot be completely released, which can be seen in Figure 5a without strain exerting. It also can be seen that at the initial stage, the average stress of the system increases linearly with the strain in the range of the strain 0–2%, as shown in the ab section of the SS curve. This is owing to the dislocation of the GB climbing along the GB, during which the deformation of the sample belongs to the elastic deformation [74]. After that, the stress increases slowly in the range of the strain 2–3.5% in the bc section of the SS curve. This indicates that the dislocation in the SGB begins to move toward two sides of the GB by gliding, which means the plastic deformation occurs obviously in this case. When the strain reaches the range of 3.5–5.0%, the stress decreases in the ce section of the SS curve and the strain softened occurs. According to Reference [74,75,76], the formation of the mature localization deformed band (grain) can be considered as the reason for the strain softened. When the strain reaches the range of 5.0–6.0%, the average stress for the system increases again in the ef section of the curve. This means that the dislocation of the two rows of the SGB of the two localization deformed bands (grain) moving toward each other interact by twisting under a resolved shear strain [62] between the two dislocations approaching each other. After the strain reaches a value greater than 6%, owing to annihilation of the dislocation in the SGB and to release of a part of the stored strain energy in the dislocations, the stress begins to decline rapidly and reaches minimum value at the strain 7%. Therefore, the transient soften-recovery process occurs at about 6–7% of strain. This indicates that under the condition of the deviatoric deformation, the coordinated movement of the GB dislocations can cause significant changes in the internal stress state of the system. Since the whole process is of only the dislocation migration without any multiplication of the dislocations, there is no any obvious hardening process for the deformation under the biaxial strain. Here, the deformation and extension of the local deformation zone are different from the local shear deformation band in metallic glass reported in Reference [74,75,76], and also different from Lűders band (LB) [77,78,79,80] at low temperature. The deformation and extension of the local deformation zone in the present paper is the result of the change of the orientation of the crystal local lattice through collective cooperative slipping of the dislocations of the GBs, while the LB is a coherently propagating mode of plastic shear with a front parallel to the primary slip planes at low temperature, which can be regarded as a solitary plastic wave that propagates at the constant stress accompanying a sudden rise of the local strain rate due to rapid dislocation multiplication [78]. Hähner [77] has pointed out that the instability of the plastic deformation that leads to anomalous stress–strain curve and to the localization of plastic strain, which may result from various underlying microscopic processes and a macroscopic deformation condition. According to the classification by Estrin and Kubin [81], the stability of the *M* type is due to that the local lattice orientation becoming unstable to activate the slip system. Here, the nucleation of the deformation band (grain) is some related to the GB splitting to activate the slip system under the strain, and the deformation band extension is related to the change of the local lattice orientation due to the instability of the orientation [82].

According to the criterion [74] for the localized shear bands, the explanation for the formation of the localized deformation zone on the basis of the SS curve in Figure 8 can be given as following. The localized deformation zone starts appearing at the strain labeled “c”, i.e., the maximum stress of the SS curve, i.e., the plastic deformation starts obviously at this strain. When the sample is strained to the point marked “e” in SS curve, one mature deformed zone is formed. Thus, the strain energy that is spent during deformation of the sample from point “c” to “e” is the energy required to the formation of the deformed grain with higher localized strain energy, accompanying with the high-density ensembles of the mobile lattice dislocation. Before the formation of a localized deformation zone (grain), the energy dissipated area under the SS curve up to point “e” can be partitioned into two parts labeled A and B. The energy in region A contributes to the local inelastic deformation and dissipates throughout the whole sample. Therefore, the energy that gets spent during the formation of the mature localized deformation zone in region B can be defined the energy as *U*_sb_. The red arrows in the SS curve as shown in Figure 8 indicate the starting and the ending strains, i.e., the point “c” and “e” can be used to estimate the values of *U*_sb_ for the mature localized deformation zone. We consider that the localization of the plastic flow through the formation of the localized deformation zone occurs if and only if the total energy that is restored in the system at the onset of plastic deformation *U*_p_ is equal or larger than *U*_sb_. If *U*_p_ < *U*_sb_, the strain energy in the area is insufficient to cause the localization, therefore, the localized deformation zone does not occur. For the e-f-g sections of the SS curve in Figure 8, the localized deformation zone transforms into non-localized deformation.

### 3.4. The Law of the Deformed Grain Growth

**Figure 9:**
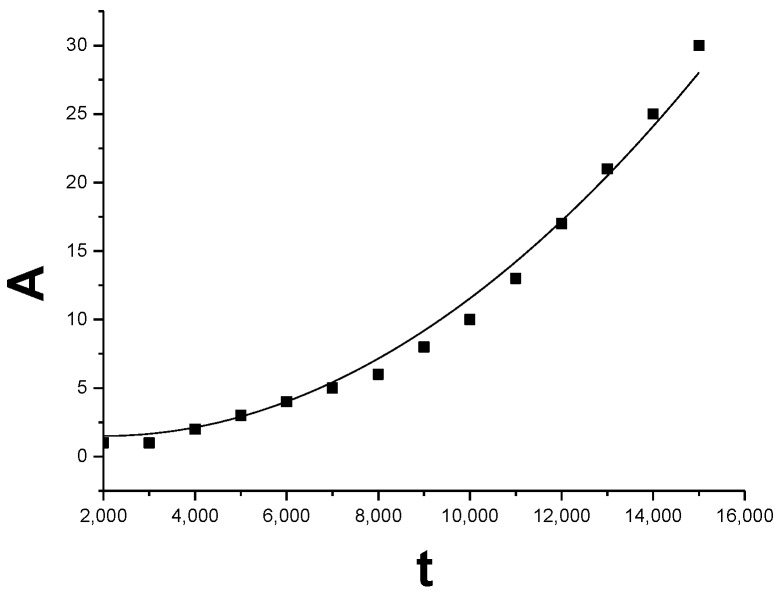
Growth curve of the area *A* of the deformed grain *vs* time *t* by the PFC simulation.

For the extension of the deformed grain, the curve of the area of the deformed grain growth (DGG) changing with time, which is gotten by PFC simulation, is shown in Figure 9. The deformed grain with 0° orientation consumes the original grains with 4° or −4° orientation to extension. This process of the DGG is the collective glide of the dislocation of the SGB, accompanying with the high strain energy zone extending toward the low strain energy zone in the system shown in Figure 5b–e. Following the conventional formula [32] of the grain growth, we express the formula of the extension of the area *A* by the strain driving as below:

(11) $$A-A_{0}=\alpha \times t^{\beta }\;$$

We obtain the formula of the DGG by fitting the data of the PFC simulation shown in Figure 9 as below

(12) $$\Delta A=A-1.5=1.56\times 10^{-5}\times t^{2.0}\;$$

where the growth coefficient *α* = 1.56 × 10^−5^, the initial constant *A*_0_ = 0.15, and the time index *β* = 2.0. The area Equation (12) of the DGG is consistent with the rule of the time square *t*^2^. Noticing the way of the SGB migration in Figure 4, the area *A* can be written as *A* = *Lx*, where *L* is the width of the sample in a constant. Therefore, the DGG along *x* direction in the length formula can be rewritten as

(13) $$\Delta D\;=x-x_{0}=\frac{\alpha }{L}\times t^{2.0}\;$$

Transforming the area extension into the length extension of the grain, we can get the speed of the growth along *x* direction is

(14) $$v_{\mathrm{GB}}=\frac{dx}{dt}=\frac{2\alpha }{L}t\;$$

It can be seen from Equation (14) that the speed of the extension along the *x* direction is a linear increase with time and the DGG is in the state of the acceleration motion. In the literature [20,30,73], the grain growth driven by the constant stress is simulated and analyzed, and the law of the grain growth is also obtained, in which the size of the grain growth increases linearly with time, i.e., the time index *β* = 1. However, the time index *β* in Equation (13) of the grain growth under the curvature [32] is about *β* = 1/2, which is different from the mechanism of the grain growth driven by the external force. Kill et al. [83] reported that the growth index of the NC Fe obeys the index law *β* = 1, while for the metal Fe of coarse crystal, its grain growth index rule obeys the index rule *β* = 1/2. This indicates that the mechanism of the grain growth is strongly dependent on the size of grain and the structure of the GB. In the case of a circular grain shrinking [44] with large or small misorientation, it obeys the *β* = 1/2 of the usually exponential rule, while for the grain shrinking of the middle misorientation, the grain growth is not subjected to the *β* = 1/2 power-law due to the change of the mechanism of the grain growth. Usually, for the mechanism of the grain growth of the curvature driven, most of them obey the *β* = 1/2 power-law [84,85,86], while on the scale of the NC grains, its growth rule deviates the *β* = 1/2 power-law to obey *β* = 1 exponential rule [83]. For the mechanism of the complex grain growth, for example, the nanotwin-assisted grain growth [87] in the NC materials also does not obey the *β* = 1/2 simple power-law.

Under the driving of the dynamic deviatoric strain in this work, the speed of the DGG for the deformation process is obviously faster than that of the grain growth under the curvature [32,88,89] in relaxation. Here, the GB dislocations slidings collectively and synchronously away from the original GB under the biaxial strain are also different than that of the report in Reference [4,5,6,7,8], in which the dislocations slide is only along the GB under the shear strain, therefore, the shape change of the grain under the shear strain only occurs without any new grain generation [6,10]. However, in this work, the original GB is split into two SGBs which move in two opposite direction under the biaxial strain and generates new DG with new orientation, which is similar to the case of the GB splitting in Reference [13]. By comparing the growth index, we can get the migration speed of the GB for the DGG which is linear with time *t* in Equation (14), and the time index is *β* = 2 in Equation (13). Obviously, the DGG under the biaxial strain is faster and accelerates with time, due to the exerted biaxial strain changing linearly with time.

### 3.5. Dynamic of the DGG

Mcreynods et al. [88] have confirmed that the dislocation climbing fully compensates for the translation of the lattice; the amount of translation predicted by coupling is compared to the number of lattice sites added or removed by dislocation climbing. According to Cahn et al. [3,5] for the case of the pure coupling (no sliding) in small angle tilt boundary, the tangential velocity *v*_t_ of the lattice is proportional to the normal velocity *v*_n_ of the GB by the misorientation angle *θ.* The driving force for GB migration and grain rotation stems from two sources: (1) internal structure, i.e., surface tension due to GB curvature and net torque due to a reduction in GB energy that triggers grain rotation; (2) externally applied stresses. Essentially, the growth rate (d*D*/d*t*) of the grain is proportional to the driving force (*P*) [20,90,91] by a mobility parameter *M*, which captures the thermally activated component of the kinetic. Here, the migration of the DGG is vertical to the GB direction, i.e., along the *x* axis direction, while the gliding direction of the GB is along the *y* axis direction. The GB dislocation moves via a combination [88] of the glide and climb, which allows the dislocation to move roughly perpendicular to the planar interface instead of along the close-packed direction [86] of the Burgers vector. The climbing mechanism also destroys or creates lattice sites to allow for the rigid body translation [86]. According to the coupling movement of the GB suggested by Cahn et al. [3,4,5], the dynamic equation of the SGB can be written as

(15) $$v_{n}=\frac{dD_{x}}{dt}=M_{ix}P_{ix}+M_{ex}P_{ex}+\beta_{x}\frac{dD_{y}}{dt}\;$$

(16) $$v_{\tau }=\frac{dD_{y}}{dt}=S_{iy}P_{iy}+S_{ey}P_{ey}+M_{iy}P_{ix}+M_{ey}P_{ex}+\beta_{y}\frac{dD_{x}}{dt}\;$$

where *D_x_* is the distance of the SGB migration and *D_y_* is the distance of the dislocation movement along the SGB and *t* is the time, and *P*_i_ is the driving force applied by internal structure, and *P*_e_ is the driving force owing to externally applied stresses. *M*_i_ and *M*_e_ are the migration parameters of the SGB migration by the driving force *P*_i_ and *P*_e_, respectively. *S* is the sliding coefficient in response to (d*D*_y_/dt) = *v*_τ_, (d*D_x_/dt*) = *v*_n_, and *β* is the coupling constant [4,5,6] of the SGB movement. For the pure STGB there are no dislocations gliding in the plane of the boundary [88]. Hence there is no dislocation mechanism for sliding, i.e., *S*_iy_ = 0 and *S*_ey_ = 0. Since the grain shape in this PFC simulation is rectangular, and also the SGB of the grain is a planar SGB, therefore, the driving force of the curvature is not considered. Because there is no the difference of dislocation density between two sides of the SGB, no the migration of the SGB is driven by dislocation density difference. Hence, we can ignore the role of internal stress, and only consider the external applied force to drive the migration of the SGB. Hence, we get the equation from Equations (15) and (16) as below

(17) $$v_{n}=M_{ex}P_{ex}+\beta_{x}v_{\tau }\;$$

(18) $$v_{\tau }=M_{ey}P_{ey}+\beta_{y}v_{n}\;$$

According to Reference [3], we have *β_x_ = β_y_ = πθ*/180 (in radian), where *θ* <8° (in degrees), then *β_x_·β_y_* <<1 for the small angle STGB, hence the *β_x_·β_y_* is a second order small amount which can be ignored here. Under the deviatoric deformation, the formula of the solution for the dynamic equations can be obtained from Equations (17) and (18), as

(19) $$v_{n}=\frac{M_{ex}P_{ex}+\beta_{x}M_{ey}P_{ey}}{1-\beta_{x}\beta_{y}}\approx M_{ex}P_{ex}+\beta_{x}M_{ey}P_{ey}=(1+\beta_{x})M_{ex}P_{ex}\;$$

(20) $$v_{\tau }=\frac{M_{ey}P_{ey}+\beta_{y}M_{ex}P_{ex}}{1-\beta_{x}\beta_{y}}\approx M_{ey}P_{ey}+\beta_{y}M_{ex}P_{ex}=(1+\beta_{y})M_{ey}P_{ey}\;$$

where we assume that *M_ex_* = *M_ey_* and *P_ex_* = *P_ey_* for simplicity. Equation (19) can be used to describe the process of the grain growth driven by the external force, while Equation (20) can be used to describe the local lattice rotation, and also the migration of the GB coupling with the applied shear force. Since the material exhibits no grain motion under thermal activation alone, the driving force must be in some form related to the work input into the system during the working operation. In the spirit of first order kinetic [20], the driving force is assumed to be linearly proportional to the rate of mechanical work done onto the system. Thus, at any moment during deformation, the driving force [20] can be written as

(21) $$P_{ex}\propto \sigma_{x}\frac{d\varepsilon_{x}}{dt}\;\mathrm{and}\;P_{ey}\propto \sigma_{y}\frac{d\varepsilon_{y}}{dt}\;$$

Then

(22) $$P_{ex}=\eta \cdot \sigma_{x}\frac{d\varepsilon_{x}}{dt}\;\mathrm{and}\;P_{ey}=\eta \cdot \sigma_{y}\frac{d\varepsilon_{y}}{dt}\;$$

where *σ_x_* and *σ_y_* are the stress during deformation, $\frac{d\varepsilon }{dt}$ is the strain rate, and *η* is a proportional constant. The kinetic approach suggests that mobility *M*_ex_ plays an important route in grain growth. The current GB mobility *M*_e_ can be expressed in an Arrhenius-type equation

(23) $$M_{ex}=M_{0}\exp(-\frac{Q_{GB}}{RT})\;$$

where *R* is the gas constant, *M*_o_ a constant and *Q*_GB_ the activation energy for isothermal grain growth, then, the grain growth speed along *x* direction is

(24) $$\begin{array}{cc} v_{\mathrm{GB}} & =v_{n}=(1+\beta_{x})M_{ex}P_{ex}\\ & =(1+\beta_{x})M_{0}\exp(\frac{Q_{GB}}{RT})\sigma_{x}\cdot \eta \frac{d\varepsilon_{x}}{dt} \end{array}$$

Let Δ*x*_0_ and Δ*y*_0_ denote the length before deformation, and Δ*x* and Δ*y* are the length after deformation, which can be written as Δ*x* = Δ*x*_0_(1 + *ε_x_*) and Δ*y* = Δ*y*_0_ (1 + *ε_y_*), then we have

(25) $$\Delta x\cdot \Delta y=\Delta x_{0}(1+\varepsilon_{x})\cdot \Delta y_{0}(1+\varepsilon_{y})=\Delta x_{0}\cdot \Delta y_{0}\cdot (1+\varepsilon_{x}+\varepsilon_{y}+\varepsilon_{x}\varepsilon_{y})=\Delta x_{0}\cdot \Delta y_{0}\;$$

Under the constant area condition of Equation (6), we can get the result from Equation (25) as below

(26) $$(1+\varepsilon_{x}+\varepsilon_{y}+\varepsilon_{x}\varepsilon_{y})=1\;$$

where *ε_x_* and *ε_y_* is a small amount, i.e, *ε_x_* and *ε_y_* << 1, then *ε_x_*·*ε_y_* is the second order small amount and can be ignored. Then we have *ε_x_* + *ε_y_* = 0, i.e., *ε_x_* = −*ε_y_*. Under this condition of the deformation, the deviatoric deformation with the constant area in two dimensions occurs.

For a plane strain, we here have the relationship [91] between the strain and stress

(27) $$\varepsilon_{x}=\frac{1-\mu^{2}}{E}(\sigma_{x}-\frac{\mu }{1-\mu }\sigma_{y})\;$$

(28) $$\varepsilon_{x}=\frac{1-\mu^{2}}{E}(\sigma_{x}-\frac{\mu }{1-\mu }\sigma_{y})\;$$

(29) $$\varepsilon_{y}=\frac{1-\mu^{2}}{E}(\sigma_{y}-\frac{\mu }{1-\mu }\sigma_{x})\;$$

where *μ* is the Poisson’s ratio, and *E* is the Young’s modulus. When *ε_x_* = −*ε_y_* is satisfy, we can have σ*_x_* = −σ*_y_* from these formula above, therefore, we get *ε_x_* = ((1 + *μ*)/E)σ*_x_* and *ε_y_* = −((1 + *μ*)/E)σ*_x_*, or σ*_x_* = (E/(1 + μ))*ε_x_* and σ*_y_* = −σ*_x_* = −(E/(1 + μ))*ε_x_*.

Since the DGG and the migration of the SGB is along the x direction, then the rate of the DGG by the driving force can be written from Equation (24) as

(30) $$\begin{array}{cc} v_{GB} & =\frac{dD_{x}}{dt}=(1+\beta_{x})M_{ex}P_{ex}=\eta (1+\beta_{x})M_{ex}\sigma_{x}\frac{d\varepsilon_{x}}{dt}=\eta (1+\beta_{x})M_{ex}\cdot \frac{E}{1-\mu }\varepsilon_{x}{\dot{\varepsilon }}_{x}\\ & =(1+\beta_{x})M_{ex}\frac{\eta E}{1-\mu }{\left({\dot{\varepsilon }}_{x}\right)}^{2}\cdot t \end{array}$$

where $\varepsilon_{x}={\dot{\varepsilon }}_{x}\cdot t$, ${\dot{\varepsilon }}_{x}$ is the strain rate. It can be seen that the *dD_x_*/*dt* is linear increasing with time in Equation (30), and the DGG is accelerated under the strain. Thus, the distance of the migration of the GB of the deformed grain is given as

(31) $$D_{x}=\frac{1}{2}(1+\beta_{x})M_{ex}\frac{\eta E}{1-\mu }{\left({\dot{\varepsilon }}_{x}\right)}^{2}\cdot t^{2}=\frac{1}{2}(1+\beta_{x})M_{ex}\frac{\eta E}{1-\mu }{\left(\varepsilon_{x}\right)}^{2}\propto {\left(\varepsilon_{x}\right)}^{2}\;$$

The rule of the DGG given in Equation (31) is agreement with Equation (13) obtained by fitting the results of the PFC simulation. It can be seen in Equation (31) that the shape and size of the deformed grain can be controlled through adjusting the strain *ε*_x_. Comparing the Equations (14) and (30), we can get

(32) $$\alpha =(1+\beta_{x})M_{ex}\cdot \frac{\eta \cdot E\cdot L}{1-\mu }\cdot {\left({\dot{\varepsilon }}_{x}\right)}^{2}\propto {\left({\dot{\varepsilon }}_{x}\right)}^{2}\;$$

where the parameter *α* is related to the *M*_e_, *E*, *L*, *ν*, and ${\dot{\varepsilon }}_{x}$. We employ the GB mobility for pure Cu metal [73], *M*_0_ = 2.5 × 10^−6^(m^4^/Js), Possion’s ratio *μ* = 0.308, the coupling constant *β*_x_ = *πθ*/180 = 0.07, *θ* = 4°, *ε* = 7%, $\dot{\varepsilon }$= 6 × 10^−6^, *E* = 128 GPa, *η* = 9 × 10^−6^ (s), high temperature *T* = 1050 K for temperature parameter *r* = 0.10 [52] of the PFC simulation, then we get the speed of the GB migration of Cu metal *V*_GB_ = 1.83 m/s, which is very close to the results of molecular dynamic simulation [60,71,92].

According to the Equation (31), the approximate formula for the strain energy of the system under a deviatoric deformation can be obtained from Figure 5a,b as below

(33) $$\Delta E=h\cdot D_{x}\approx \varpi \cdot \varepsilon^{2}\;$$

where *h* is the increment of the energy platform height in Figure 5, and *D*_x_ is the width of the platform. By connecting the Equations (31) with (33), then we have $\varpi =\frac{1}{2}h\cdot (1+\beta_{x})\cdot M_{e}\cdot \frac{\eta E}{1-\mu }$. It can be seen that the Equation (33) is consistent with the rule of Equation (9) for the strain energy with item *ε*^2^ under a small strain condition.

### 3.6. Dynamic of Dislocation of STGB under Deviatoric Deformation

**Figure 10:**
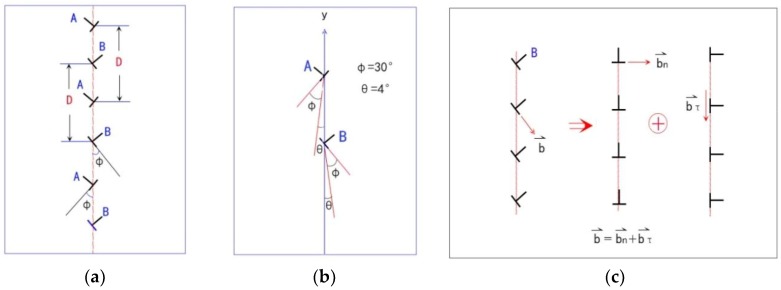
Schematic of GB dislocation: (**a**) Two type dislocations A and B arranging alternatively in the STGB, (**b**) Tilt angle *θ* of the STGB and atomic arrangement orientation *Φ* of the grain, (**c**) The dislocation vector ${\vec{b}}_{}$ decomposition: one ${\vec{b}}_{n}$ is the vertical to the GB, which forms a similar structure of the sessile extrinsic grain boundary dislocation (EGBD) [94] and leads to the GB migration and grain growth, and the other ${\vec{b}}_{\tau }$ is parallel to the GB, which forms a similar structure of the glissle EGBD and leads to the dislocation motion along the GB and the grain orientation change.

The motion of the GBD of the circular bi-crystal under a stress has been reported in Reference [3], here in this work the complicated dislocation arrangement of the straight STGB of bi-crystal with two sets of the dislocation structure under deviatoric deformation as shown in Figure 2b and Figure 10a will be discussed. Owing to the structure of the small angle STGB, the spacing of the two sets of the dislocations [63] is *D*_1_ = *D*_2_ = *D* = *b*/*θ*, $\vec{b}$ is the Burgers vector, and *θ* is the misorentation angle of the STGB, shown in Figure 10a,b. The GB is along the *y* direction, the tilt angle *θ* is ±4° by *y* axis, and the crystal orientation *Φ* is about 30° by *y* axis in Figure 10b. The GB in Figure 2b and Figure 10a is split into two SGBs as shown in Figure 4. Here we take the type of the dislocation B of the GB shown in Figure 10a as an example to discuss the motion of the dislocations under the biaxial strain. The angle between the vector of the dislocation and direction of the SGB (along *y* direction) is about *Φ* + *θ* ≈ 34°, shown in Figure 10b. The vector of the dislocation B, $\vec{b}=\;a[01\bar{1}]$, can be decomposed of two vectors, one is ${\vec{b}}_{\tau }=\frac{a}{2}[11\bar{2}]$ parallel to the SGB along the *y* direction arrangement, which forms a structure similar to the Glissile extrinsic grain boundary dislocation (EGBD) [93,94], and the other is ${\vec{b}}_{n}=\frac{a}{2}[\bar{1}10]$ perpendicular to the SGB along the *y* direction arrangement, which forms a structure similar to the sessile EGBD [94], as shown in Figure 10c. The SGB migration is that the dislocations ${\vec{b}}_{n}=\frac{a}{2}[\bar{1}10]$ in the SGB glides collectively perpendicular to the SGB direction and makes the grain grow, while that the dislocation ${\vec{b}}_{\tau }=\frac{a}{2}[11\bar{2}]$ of the GB moves along the GB and leads the lattice orientation to change in the place near the GB of the grain.

**Figure 11:**
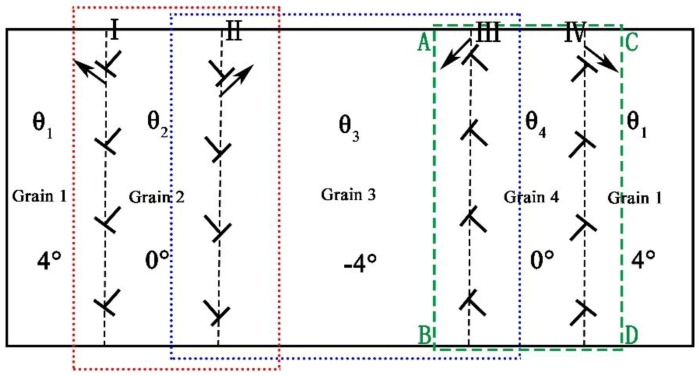
Schematic of the alternating arrangement of the dislocations of the GBs is separated into two planar SGBs under the deviatoric deformation and generates new deformed grain 2 and 4 with orientation *θ*_2_ and *θ*_4_, respectively. More detail snapshots for evolution of the dislocation of the SGB can be seen in Reference [55].

It can be seen in Figure 10c that the slip direction of the dislocation $\vec{b}=\;a[01\bar{1}]$ is parallel to the slip system (the direction of the lattice arrangement) of the sample shown in Figure 4, and takes approximately the angle 30° by *y* axis. Trautt et al. [2] has pointed out that the dislocation motion being vertical to the GB and parallel to the GB are all of the combinations of two kinds of movement of climbing and gliding in two-dimensional triangular state in the PFC simulation. Although the dislocation ${\vec{b}}_{n}$ and ${\vec{b}}_{\tau }$ of the GB moves along the direction of the normal stress *σ*_x_ and *σ*_y_ as shown in Figure 10c, the motion of the dislocations is the combination of climbing and sliding motion, instead of pure climbing or sliding motion of the dislocation, which is due to that the Burgers vector ${\vec{b}}_{n}=\frac{a}{2}[\bar{1}10]$ and ${\vec{b}}_{\tau }=\frac{a}{2}[11\bar{2}]$ are not fully parallel to the direction of the slip system. The deformed grain grows through the dislocation of the SGB gliding as shown in the red box of Figure 11, while that of the SGB sliding makes the original grain shrink in blue box of Figure 11.

The SGB migration resembles a continuous, viscous movement [92] of the dislocations under an applied persistent force. According to Reference [63], irrespective of the underlying mechanism of the GB drift velocity, the velocity *v* should be related linearly to driving force *P*, i.e., *v* = *MP*, provided that *PΩ*/*kT* <<1 (where *Ω* is the atomic volume, *k* is the Boltzmann constant and *T* the absolute temperature). The phenomenological Equation (14) indicates that under the action of the internal and external constant force, the GB motion is in uniform speed motion. The motion of the system is in stable dynamic equilibrium, while the actual dislocation motion of the GB is a process of the change of the velocity under a net force. Therefore, in order that the system can finally reach a stable equilibrium, it requires the kinetic equation should include a term of a damping force [93]. The dislocation motion, in addition to the external stress, are also affected by the damping force, which is assumed to be directly proportional to the velocity of the dislocation motion [65,93]. The dynamic of a single dislocation motion is well described by the equation of motion for a point mass in a damped medium for the stick-slip character of the dislocation movement [65]. For kinetic equation of the dislocation of the SGB for the DGG in damped medium, according to the Equation (A7a) in Appendix, under the condition of *H*_x0_ = 0, we can get the formula of the velocity along x direction as

(34) $$v_{x}\left(t\right)=\frac{{\bar{\alpha }}_{y}}{{\bar{\beta }}_{x}}\cdot t+\frac{\left[1-e^{-{\bar{\beta }}_{x}\cdot t}\right]}{{\bar{\beta }}_{x}}\left[-\frac{{\bar{\alpha }}_{y}}{{\bar{\beta }}_{x}}\right],\;$$

where ${\bar{\alpha }}_{y}=(\alpha_{y}/m)\cdot b\cdot \sin\theta ,{\bar{\beta }}_{x}=\beta_{x}/m,\;{\bar{H}}_{x_{0}}=H_{x_{0}}/m=\;0$. When t→+∞, we get

(35) $$v_{x}\left(t\right)=\frac{{\bar{\alpha }}_{y}}{{\bar{\beta }}_{x}}\cdot t-\frac{1}{{\bar{\beta }}_{x}}\left[\frac{{\bar{\alpha }}_{x}}{{\bar{\beta }}_{x}}\right]=\frac{{\bar{\alpha }}_{y}}{{\bar{\beta }}_{x}}(t-\frac{1}{{\bar{\beta }}_{x}})\approx \frac{{\bar{\alpha }}_{y}}{{\bar{\beta }}_{x}}\cdot t\;$$

For a long time evolution of the DGG, we approximately have $v_{x}(t)\approx \frac{{\bar{\alpha }}_{y}}{{\bar{\beta }}_{x}}\cdot t$, which is an good agreement with Equations (14) and (30). This indicates that the velocity of the dislocations is linear with time *t* in a stable state under a damped force. We can get the migrated distance of the SGB in the initial condition *x*(t = 0) = 0 from Equations (A8a) and (A8b) in Appendix as below:

(36) $$x\left(t\right)=\frac{{\bar{\alpha }}_{y}}{2{\bar{\beta }}_{x}}t^{2}-\frac{1}{{\bar{\beta }}_{x}}\left[\frac{\alpha_{y}}{{\bar{\beta }}_{x}}\right]\left[t+\frac{1}{{\bar{\beta }}_{x}}\left(e^{-{\bar{\beta }}_{x}\cdot t}-1\right)\right]\;$$

(37) $$x\left(+\infty \right)=\frac{{\bar{\alpha }}_{y}}{2{\bar{\beta }}_{x}}\cdot t^{2}-\frac{1}{{\bar{\beta }}_{x}}\left[\frac{{\bar{\alpha }}_{y}}{{\bar{\beta }}_{x}}\right]\left[t-\frac{1}{{\bar{\beta }}_{x}}\right]\approx \frac{{\bar{\alpha }}_{y}}{2{\bar{\beta }}_{x}}\cdot t^{2},\;(t\to +\infty )\;$$

By comparing the growth index, we can see the DGG relationship in Equation (37) is linear with time square *t*^2^, and is a good agreement with Equations (11) and (31) and also the time index is *β* = 2.

For pure Cu metal, lattice constant *a* = 0.361 nm mass density *ρ* = 8900 kg·m^−3^, activation energy of the dislocation Q_GB_ = 0.23 eV, dislocation mass (per unit length of dislocation line), m = 2.4 × 10^−16^ kg·m^−1^, Burger vector b=a〈111〉/2=0.3126 nm $\beta_{dis}=\beta_{0}\exp(-\frac{Q_{dis}}{RT})$, *β*_0_ = 14.5 Pa·s, *β*_0_ is the damping coefficient at room temperature, *T* = 1050 K, Young’s modulus *E* = 128 GPa, $\alpha_{x}=\frac{E\dot{\varepsilon }}{1+\mu }$ and $\alpha_{y}=-\frac{E\dot{\varepsilon }}{1+\mu }$, $\dot{\varepsilon }=\;6\times 10^{-6}\left(s^{-1}\right)$, then we get approximately the speed of the dislocation migration of Cu metal ***v***_dis_ = 1.98 (m/s) at *ε* = 7%, which is close to the results of the previous section.

## 4. Conclusions

(1) Under biaxial strain, the deformed grain nucleates through the original GB splitting to form the gap between two SGBs. The collective dislocation migration of the SGB is the movement of the ensembles of the mobile lattice dislocations, which causes the localized strain energy and the localized plastic flow. This is owing to the change of the crystal lattice orientation due to the instability of the orientation under the biaxial strain.

(2) The deformed grain is of new orientation and high localized strain energy. With the deformed grain extending, the higher strain energy zone of the deformed grain extends through the SGB migration into the lower strain energy zone of the original grain. The SGB is the boundary line between the high localized strain energy zone of the deformed grain and low strain energy zone of the original grain.

(3) The DGG is that the SGB propels toward the interior of the original grain, and the speed of propulsion of the DGG is linear to the time, and the law of the DGG follows *β* = 2 of the time index of the growth. The process of the DGG is faster than that of the growth of the grain under the curvature.

## Abbreviations

PFC:	Phase field crystal
DGG:	Deformed grain growth
GBD:	Grain boundary dislocation
GBS:	Grain boundary sliding
CDM:	Cooperative dislocation movement
DG:	Deformed grain
OG:	Original grain
STGB:	Symmetric tilt grain boundary
SGB:	Sub-grain boundary
LB:	Lűders band
SS:	Stress-strain
NC:	Nanocrystalline
EGBD:	Extrinsic grain boundary dislocation

## Appendix A. Dynamic Equation of Dislocation for STGB Migration and DGG

Considering the applied strain shown in Figure 1, in which the *σ*_x_ and *σ*_y_ are the normal stress components, respectively, we can get the stress tensor of the external force field, and write as

(A1) $$\vec{\sigma }=\left(\begin{array}{ccc} \sigma_{x} & 0 & 0\\ 0 & -\sigma_{y} & 0\\ 0 & 0 & 0 \end{array}\right)\;$$

According to the Reference [63] the force of the stress tensor which is subjected to the dislocation can be written as

(A2) $$\begin{array}{l} \vec{F}=\vec{b}\cdot \hat{\sigma }\times d\vec{l}=\left(b_{x},\;b_{y},\;b_{z}\right)\left\{\sigma_{\mathrm{ij}}\right\}\left(\begin{array}{l} i\\ j\\ k \end{array}\right)\times d\vec{l}=\left(b\sigma_{x}\cos\phi \cdot \vec{i}+b\sigma_{y}\sin\phi \cdot \vec{j}+0\right)\times \left(\begin{array}{l} 0\\ 0\\ 1 \end{array}\right)\\ \;=b\left|\begin{array}{ccc} \;i & \;j & \;k\\ \sigma_{x}\cos\phi & \sigma_{y}\sin\phi & \;0\\ \;0 & \;0 & \;1 \end{array}\right|=\left(b\sigma_{y}\sin\phi ,-b\sigma_{x}\cos\phi ,0\right) \end{array}$$

From the Equation (A2), the applied force component along x and y direction exerting on the dislocation can be written as

(A3a) $$F_{x}=\sigma_{y}b\sin\phi =\alpha_{y}\cdot b\cdot t\sin\theta \;=\left(\alpha_{y}\cdot b\sin\phi \right)t\;$$

(A3b) $$F_{y}=-\sigma_{x}b\cos\phi =-\alpha_{x}\cdot b\cdot t\cdot \cos\theta \;=-\left(\alpha_{x}\cdot b\cdot \cos\phi \right)t\;$$

where $\sigma_{x}=\alpha_{x}\cdot t$ and $\sigma_{y}=\alpha_{y}\cdot t$, *α*_x_ and *α*_y_ are the proportional constant, which are $\alpha_{x}=\frac{E\dot{\varepsilon }}{1+\mu }$ and $\alpha_{y}=-\frac{E\dot{\varepsilon }}{1+\mu }$, respectively, and *E* is Young’s modulus and $\dot{\varepsilon }$ is the strain rate.

The Equations (17) and (18) indicates that under the action of the internal and external constant force, the GB motion is uniform motion. The motion of the system is in a stable state, while the actual dislocation motion of the GB is the process of the acceleration of the velocity under the net force exerting. Therefore, it will be required that the kinetic equation should include a term of a damping force, in order that the motion can finally reach a stable motion. The dislocation motion, in addition to the external stress, is also affected by the damping force [93], which is assumed to be directly proportional to the velocity of the dislocation motion. The reason for the damping is that the dislocation is in the state of viscous motion [93]. The atomic potential field of the lattice can be approximately considered as the uniform potential field, and the periodicity of the potential field is ignored, which is the resistance for dislocation motion. Under the action of the constant external stress, the separation of two sets of the dislocations in the GB occurs. This process is similar to that of partial dislocation separation under applied stress [95]. The two sets of the GB are separated to form two SGBs. The region between the two separated GBs is the completely elastic-plastic zone [70], which can exert an attractive force on the separated GBs [95] by a steady deformation flow, to dray the SGBs to go back. We use the *H*_x0_ to denote the drag force on the SGB, which direction is vertical to the SGB, i.e., along $\left[\bar{1}10\right]$ direction of *x* axis.

According to the analysis for the forces exerting on the dislocation in the previous part above, the dynamic of a single motion dislocation is well described by the equation of the motion for a point mass in damped medium [65,93,96], and the dynamic equation can be written as [93],

(A4a) $$m\frac{d^{2}x}{dt^{2}}=F_{x}\left(t\right)-\beta_{x}\frac{dx}{dt}+H_{x_{0}}=\left(\alpha_{y}\cdot b\cdot \sin\phi \right)t-\beta_{x}\cdot \frac{dx}{dt}+H_{x_{0}}\;$$

(A4b) $$m\frac{d^{2}y}{dt^{2}}=F_{y}\left(t\right)-\beta_{y}\frac{dy}{dt}=\left(-\alpha_{x}\cdot b\cdot \cos\phi \right)t-\beta_{y}\cdot \frac{dy}{dt}\;$$

Supposing the damping coefficient *β* can be expressed in an Arrhenius-type equation

(A5) $$\beta_{dis}=\beta_{0}\exp(-\frac{Q_{dis}}{RT})\;$$

where *Q*_dis_ is activation energy for dislocation movement, and *β*_0_ is the damping coefficient at room temperature. Dynamic equation of the dislocation of the grain growth along *x* direction from Equation (A4a), we have

(A6a) $$\frac{d^{2}x}{dt}={\bar{\alpha }}_{y}\cdot t-{\bar{\beta }}_{x}\cdot \frac{dx}{dt}+{\bar{H}}_{x_{0}}\;$$

where ${\bar{\alpha }}_{y}=\;\alpha_{y}\cdot b\cdot \sin\theta ,{\bar{\beta }}_{x}=\beta_{x}/m,\;{\bar{H}}_{x_{0}}=H_{x_{0}}/m\;$. Let $\frac{dx}{dt}=v_{x}$, then the equation can be written as

(A6b) $$\frac{dv_{x}}{dt}+{\bar{\beta }}_{x}\cdot v_{x}-{\bar{\alpha }}_{y}\cdot t-{\bar{H}}_{x_{0}}=0\;$$

under the initial condition, $v_{x}(t=0)=0,$ the velocity solution can be written as

(A7a) $$v_{x}\left(t\right)=\frac{{\bar{\alpha }}_{y}}{{\bar{\beta }}_{x}}\cdot t+\frac{\left[1-e^{-{\bar{\beta }}_{x}\cdot t}\right]}{{\bar{\beta }}_{x}}\left[H_{x_{0}}-\frac{{\bar{\alpha }}_{y}}{{\bar{\beta }}_{x}}\right]\;$$

when t→+∞, we get

(A7b) $$v_{x}\left(t\right)=\frac{{\bar{\alpha }}_{y}}{{\bar{\beta }}_{x}}\cdot t+\frac{1}{{\bar{\beta }}_{x}}\left[H_{x_{0}}-\frac{{\bar{\alpha }}_{x}}{{\bar{\beta }}_{x}}\right]\;$$

During the initial stage, the solution satisfies $v_{x}(t)\approx H_{x0}\cdot t$, while for a long time we have a stable form of the speed $v_{x}(t)\approx \frac{{\bar{\alpha }}_{y}}{{\bar{\beta }}_{x}}\cdot t$. The speed is linear with time *t*. Under the initial condition x(t=0)=0, we can get *x*(*t*) from Equation (A7a) by integral,

(A8a) $$x\left(t\right)=\frac{{\bar{\alpha }}_{y}}{2{\bar{\beta }}_{x}}t^{2}+\frac{1}{{\bar{\beta }}_{x}}\left[H_{x_{0}}-\frac{\alpha_{y}}{{\bar{\beta }}_{x}}\right]\left[t+\frac{1}{{\bar{\beta }}_{x}}\left(e^{-{\bar{\beta }}_{x}\cdot t}-1\right)\right]\;$$

(A8b) $$t\to +\infty ,x\left(+\infty \right)=\frac{{\bar{\alpha }}_{y}}{2{\bar{\beta }}_{x}}\cdot t^{2}+\frac{1}{{\bar{\beta }}_{x}}\left[H_{x_{0}}-\frac{{\bar{\alpha }}_{y}}{{\bar{\beta }}_{x}}\right]\left[t-\frac{1}{{\bar{\beta }}_{x}}\right]\;$$

It can be seen that *x*(t) is proportional to *t*^2^ when time is long enough. Dynamic equation of the dislocation of the grain growth along y direction, from Equation (A4b), we have

(A9a) $$\frac{d^{2}y}{dt^{2}}=\frac{F\left(t\right)}{m}-\frac{\beta_{y}}{m}\frac{dy}{dt}=-{\bar{\alpha }}_{x}\cdot t-{\bar{\beta }}_{y}\cdot \frac{dy}{dt}\;$$

where ${\bar{\alpha }}_{x}=\frac{\alpha_{x}}{m}b\cos\theta ,{\bar{\beta }}_{y}=\frac{\beta_{y}}{m}.$ Set $\frac{dy}{dt}=v_{y}$, then we have

(A9b) $$\frac{dv_{y}}{dt}+{\bar{\beta }}_{y}\cdot v_{y}+{\bar{\alpha }}_{x}\cdot t-{\bar{H}}_{y_{0}}=0\;$$

Under the initial condition $v_{y}\left(t=0\right)=0$, the velocity solution can be gotten

(A10a) $$v_{y}\left(t\right)=-\frac{{\bar{\alpha }}_{x}}{{\bar{\beta }}_{y}}\cdot t+\frac{1}{{\bar{\beta }}_{y}}\left[\frac{{\bar{\alpha }}_{x}}{{\bar{\beta }}_{y}}\right]\left(1-e^{-{\bar{\beta }}_{y}\cdot t}\right)\;$$

When

(A10b) $$v_{y}\left(\infty \right)=-\frac{{\bar{\alpha }}_{x}}{{\bar{\beta }}_{y}}\cdot t+\frac{1}{{\bar{\beta }}_{y}}\left[\frac{{\bar{\alpha }}_{x}}{{\bar{\beta }}_{x}}\right]\approx -\frac{{\bar{\alpha }}_{x}}{{\bar{\beta }}_{y}}\cdot t,\;t\to \infty \;$$

Under the initial condition y(t=0)=0, we get the solution by integral as,

(A11) $$\;y\left(t\right)=-\frac{{\bar{\alpha }}_{x}}{2{\bar{\beta }}_{y}}\cdot t^{2}+\frac{1}{{\bar{\beta }}_{y}}\left[\frac{{\bar{\alpha }}_{x}}{{\bar{\beta }}_{y}}\right]\left[t+\frac{1}{{\bar{\beta }}_{y}}\left(e^{-{\bar{\beta }}_{y}\cdot t}-1\right)\right]\;$$

(A12) $$\;y\left(+\infty \right)=-\frac{{\bar{\alpha }}_{x}}{2{\bar{\beta }}_{y}}\cdot t^{2}+\frac{1}{{\bar{\beta }}_{y}}\left[\frac{{\bar{\alpha }}_{x}}{{\bar{\beta }}_{y}}\right]\left[t-\frac{1}{{\bar{\beta }}_{y}}\right],\;t\to \infty \;$$

It can be seen that *y*(t) is proportional to *t*^2^ when time is long enough.
